# The Mediating Role of Parenting Style in the Relationship between Parents’ Openness to Different Ways of Thinking and Child Anxiety

**DOI:** 10.3390/children10091564

**Published:** 2023-09-17

**Authors:** Adele Zeevi-Cousin, Osnat Lavenda

**Affiliations:** School of Social Work, Ariel University, Ariel 40700, Israel

**Keywords:** parental style, anxiety, child wellbeing, parenting

## Abstract

The quality of parent–child relationships plays a significant role in the development of child anxiety, especially regarding aspects of parental control, intrusive behavior, and a lack of warmth. Nevertheless, the underlying mechanisms of these parenting behaviors that are associated with the risk of child anxiety have yet to be revealed. The present study aims to examine the contribution of a cognitive aspect of parenting, i.e., openness to different ways of thinking, to the development of child anxiety through its impact on parenting style. A sample of 300 Israeli parents (72% women) over the age of 18 (M = 38.8, SD = 6.2), with at least one child over the age of 6 (M = 13.3, SD = 5.5 of oldest child), was recruited through social media platforms. Participants provided demographic information and filled out self-reported questionnaires dealing with child anxiety (using the Child Behavior Checklist), openness to different ways of thinking (using the Interpersonal Reactivity Index), and parenting style (using the Parental Behavior Inventory). The analysis confirmed the mediation role of hostile/coercive parenting style in the association between parental openness to different ways of thinking and child anxiety. However, the association between supportive/engaged parenting and child anxiety was non-significant. Apparently, openness to different ways of thinking allows for parents to consolidate parenting that does not resort to coercive and hostile behaviors, control, obedience, and severe strictness. As a result, the child develops self-regulation and coping mechanisms that reduce the risk for developing anxiety.

## 1. Parental Style and Openness to Different Ways of Thinking as Predictors of Child Anxiety

Anxiety disorders are one of the most common psychiatric problems experienced by children [1,2,3]. Its prevalence is about 10% of child and adolescent population [4]. Anxiety is defined as a negative mood affected by negative thoughts and perceptions and arises in response to anticipation of a future threat. It is associated with impaired children functioning in many different areas [4]. For example, children with clinical and subclinical symptoms of anxiety have difficulties in coping with regular developmental challenges [5], and have difficulties in social relationships [6].

The onset of anxiety disorders is often in early childhood [7], and if left untreated, are associated with increased risk of anxiety, depression, drug use, and suicide attempts in old age [8,9,10]. In addition, untreated anxious children grow up to be adults with a high probability of absenteeism in the workplace, unemployment, repeated visits to medical specialists, and more frequent illness [11].

Although studies among twins and adopted children suggest that 25–35% of the variance in child anxiety can be attributed to genetic effects [12], the remaining unexplained variance is attributed, at least in part, to environmental factors, such as the characteristics of the parent–child relationship. The quality of parent–child relationship has significant impact on the individual’s abilities, resilience, flexibility, and mental wellbeing [13]. Indeed, theorists suggest that child anxiety is caused and influenced by parent–child interactions during childhood [14,15] through basic learning mechanisms and through effects of parents’ responses to the child’s coping strategies and emotional regulation. Theoretical models of anxiety disorders not only indicate an association between childhood anxiety and parental behaviors such as overprotection, lack of warmth and high amounts of criticism [4], psychological control and intrusive behavior [15], but they also emphasize the impact of parenting on the development, preservation, and intensification of anxiety [4,15,16].

The present study aims to better understand this relationship between parenting and child anxiety. Particularly, we examined the contribution of parent’s characteristics to explain child anxiety. The rationale of the present study stems from previous findings indicating that parenting styles are associated with child anxiety (see, for example, [17,18]). The present study uses the typology of Lovejoy et al. [19] for the two broad dimensions of parenting: supportive/engaged and hostile/coercive parenting. Supportive/engaged parenting reflects the parent’s acceptance of the child and provides autonomy and positive reinforcement to the child [20] through affection, warmth, shared activities, and emotional and instrumental support. The parent responds to the child’s needs, is committed to the child’s wellbeing, shows enthusiasm for their achievements, and demonstrates sensitivity when the child is distressed. Low levels of support and involvement were found in families with various psychological problems, including parental depression [19].

Hostile/coercive parenting has been defined as behavior characterized by criticism, high levels of parental control and rejection [20], negative influence or indifference towards the child, which may include the use of coercion, threat, or physical punishment [21]. High levels of hostility and coercive behaviors have been found in families with a wide range of psychological problems, including child abuse, aggression, and ADHD [19]. These extreme ends of parenting styles, e.g., overcontrolling, overprotecting, low levels of warmth and intrusive behaviors, are associated with adverse effects in children and adolescents, including behavioral problems, stress, and anxiety [15,21,22].

Since the literature indicates that parenting styles greatly impact child anxiety—particularly hostile/coercive parenting, of which reduces the child’s autonomy and sets rigid boundaries, regardless of the child’s needs and/or desires [2]—it was important to examine what aspect of the parent’s characteristics might lead to such parenting styles. We assumed that a cognitive mechanism which prevents the parent from understanding the child’s perspective might shed light on the impact parenting has on the child’s wellbeing. Therefore, we examined the association between parent’s openness to other ways of thinking and child anxiety through parenting styles.

Openness to different ways of thinking, also known as “perspective perception” [23,24], reflects the tendency to understand and consider other people’s perspectives or psychological points of view and is one of the cognitive dimensions of empathy [24], which involves the consideration of multiple arguments instead of the first argument that comes to mind [25]. Openness to different ways of thinking includes the attempt to understand the preferences and needs of the other [26], and leads to the respectful treatment of others [25]. It is, therefore, a significant parental characteristic that influences parenting behaviors. Furthermore, studies indicate that openness to different ways of thinking is positively associated with empathic responses and is negatively associated with behaviors that form the basis for hostile/coercive parenting [27].

The positive effect of openness to different ways of thinking was also evident in the context of child outcomes. It was found that children of parents who emphasize obedience, on the one hand, and are open to different ways of thinking, on the other, will likely develop high levels of interpersonal and communication skills, high levels of psychological-, emotional-, and even physical-wellbeing, due to their emotional and cognitive flexibility and ability to make informed, healthy life choices as well as coping mechanisms [28,29]. In contrast, parents who always consider their opinion as correct and tend to refer to others’ opinions as deficient or illegitimate [25], and who emphasize obedience and use punitive means, have children who experience difficulties in their social-, communication-, and information processing-skills, as well as anxiety and depression [29,30].

Considering all the above, the quality of the parent–child relationship plays a significant role in the development of child anxiety [4]. To the best of our knowledge, although the association between parenting style and child anxiety has been well-established in the literature, the association between openness to different ways of thinking and child anxiety was never studied in the context of parenting styles. The preset study seeks to fill in this gap in knowledge by examining the direct relationship between parental openness to different ways of thinking and anxiety among children, as well as the indirect relationship between these variables through parenting styles, with the understanding that parental openness to different ways of thinking shapes parenting, which in turn affects the level of child anxiety. Therefore, we hypothesized that (a) parental openness to different ways of thinking will be negatively associated with child anxiety and hostile/coercive parenting, but positively associated with supportive/engaged parenting; (b) supportive/engaged parenting will be negatively associated with child anxiety, while hostile/coercive parenting will be positively associated with child anxiety; and (c) parenting styles will mediate the association between openness to different ways of thinking and child anxiety.

## 2. Method Procedure and Participants

A cross-sectional design was used to examine the study’s hypotheses with a convenience sampling method. Following the approval of the Ethics committee at the authors’ University (approval No. AU-OL-20181115), a link to an electronic survey was disseminated through social media platforms (Facebook and WhatsApp). Participants were informed about the aim of the survey, the issues that the survey deals with, and the anonymous nature of the survey. They were then asked to sign an informed consent form.

Data collection started on 1 December 2018, and ended on 28 February 2019. The inclusion criteria where Jewish Israeli parents above the age of 18, with at least one child above the age of 6. This age was selected since early childhood requires special intensive care from parents. Such intensive care was an intervening factor that we excluded. A total of 362 parents participated in the survey; among them, 62 participants did not finish the survey and their records were omitted from the database. Therefore, the study’s sample included 300 Jewish parents over the age of 18 who live in Israel with at least one child above the age of 6 (Please see Table 1 for more details).

## 3. Study’s Measures

### 3.1. Demographics

The participants were asked to report their age, gender (male, female), marital status (married or in a committed relationship, separated, divorced, widowed), subjective economic status (e.g., using the item “How would you define your economic status?”, participants were asked to rate their status as: not good at all, not so good, pretty good, good, very good), education level (high school diploma, vocational diploma, bachelor’s degree, master’s degree or higher), employment status (employed, not employed), the number of children, and the age of their youngest and oldest child.

### 3.2. Child’s Anxiety

The children’s anxiety was assessed using the Anxiety/Depression subscale of the Child Behavior Checklist (CBCL), developed by Achenbach [31,32]. This 13-item subscale assesses the anxious and depressive symptomatology of a child, based on the parent’s report. Each item is coded as 0 = “Not true”, 1 = “somewhat or sometimes true”, or 2 = “very or often true”. The total score was an average score of all 13 items. The CBCL is an extensively used measure for clinical and research purposes in Israel, where the present study was conducted. Previous studies have indicated high internal consistency in the Israeli context (see, for example, [33,34,35]). Cronbach’s alpha in the present study was 0.81.

### 3.3. Parental Openness to Different Ways of Thinking

Parental openness to different ways of thinking was assessed using the perspective taking (PT) subscale of the Interpersonal Reactivity Index (IRI), which was developed by Davis [36]. This 7-item subscale measures the individual’s tendency to spontaneously adopt the psychological point of view of others in everyday life. Participants were asked to rate their answers on a 5-point Likert scale ranging from 1 = “Does not describe me well” to 5 = “Describes me very well”. The total score was an average score of all 7 items. The scale’s reliability was proven to be good (see, for example, [37,38]), and Cronbach’s alpha in the present study was 0.78.

### 3.4. Parenting Style

Parenting styles were assessed using the Parent Behavior Inventory (PBI), developed by Lovejoy et al. [19]. The questionnaire included 10 items assessing engaged/supportive parenting behaviors and 10 items assessing hostile/coercive parenting behaviors. Participants were asked to rate the extent to which they behave as described in each item, on a 6-point Likert scale, ranging from 1 = “Not true/I do not do that at all” to 6 = “Very true/I do that all the time”. The total score for each parenting style was an average score of the relevant items. The scale’s internal consistency was proven to be very good in the Israeli context (for example, see, [39,40]); in the present study, Cronbach’s alpha was 0.68 for the hostile/coercive parenting style and 0.88 for the engaged/supportive parenting style.

### 3.5. Statistical Analysis

A post hoc power analysis was conducted to ensure that the sample size was sufficient to detect a mediating effect, using the MedPower calculator [41]. By assuming a medium effect of 0.3 [42] for each of the two paths of the indirect effect (a, b) and a smaller effect of 0.1 for the direct path (c’), it was found that a sample of 300 participants yielded a power of 99% to detect a significant mediating effect, with an alpha of 0.05.

After eliminating outliers and partially filled-out records, scales were computed, and measures of reliability, normality and collinearity were calculated. Cronbach’s alpha, which was calculated for the scales, ranged from 0.68 to 0.88, indicating reasonable-to-good reliability. The values of skewness ranged between −1.565 and 1.074, and kurtosis ranged between −0.139 and 4.084, indicating a normal distribution of variables [43]. In addition, VIF values ranged between 1.084 and 1.299, indicating that there are no collinearity issues as well.

Pearson correlation coefficients were then computed to examine the relationships between the study’s variables (see Table 2); finally, mediation analysis was conducted using the PROCESS macro for SPSS version 3.4 [44].

## 4. Results

Table 1 summarizes the sample’s characteristics, indicating that above two thirds of the sample were women (72%), most were married or in a committed relationship (92.3%), and most had an academic degree (29.9% with a bachelor’s degree and 40.8% with a master’s degree). Most of the participants reported pretty good to very good SES (87.8%), and most of them reported that they were employed (95%). The participants’ ages ranged from 24 to 58 (*M* = 38.77, *SD* = 6.19).

**Table 1:** The sample’s characteristics.

Variable		Mean/%	*SD*
Gender	Male	28.0	
	Female	72.0	
Marital Status	Married/in a committed relationship	92.3	
	Separated	1.7	
	Divorced	4.3	
	Widow	0.7	
Education	High school diploma	9.9	
	Vocational diploma	19.4	
	Bachelor’s degree	29.9	
	Master’s degree or higher	40.8	
SES	Not good at all	2.3	
	Not so good	9.7	
	Pretty good	40.9	
	Good	30.5	
	Very good	16.4	
Employment status	Employed	95.0	
	Not employed	5.0	
Parent’s age		38.8	6.2
Number of children		2.3	1.2
Age of youngest child		4.2	3.6
Age of oldest child		13.3	5.5

The correlation coefficients, presented in Table 2, indicate that parental openness to different ways of thinking was positively and significantly associated with supportive/engaged parenting (r = 0.425, *p* < 0.001), and was negatively and significantly associated with hostile/coercive parenting styles (r = −0.278, *p* < 0.001). Child anxiety was only significantly associated with hostile/coercive parenting styles, indicating a positive association (r = 0.279, *p* < 0.001).

**Table 2:** Correlation coefficients between the study’s variables.

	Openness to Different Ways of Thinking	Supportive/Engaged Parenting	Hostile/Coercive Parenting
Openness to different ways of thinking	1		
Supportive/engaged parenting	0.425 **	1	
Hostile/coercive parenting	−0.278 **	−0.134 *	1
Child anxiety	−0.114	−0.070	0.279 **

* *p* < 0.05; ** *p* < 0.01.

**Figure 1:**
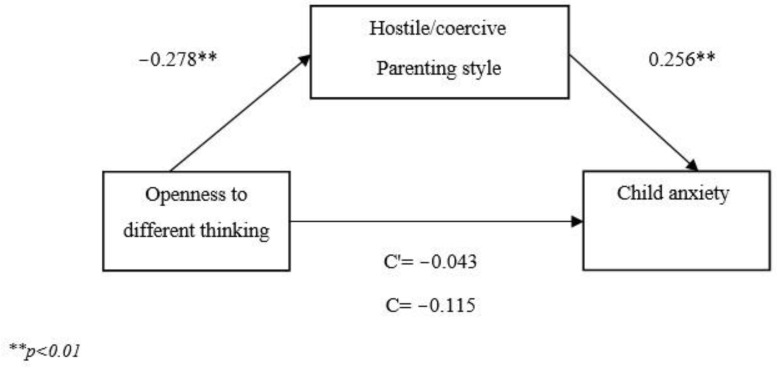
The mediating effect of hostile/coercive parenting style of the association between parental openness to different ways of thinking and child anxiety.

Since the association between child anxiety and the supportive/engaged parenting style was non-significant, the mediation model was conducted with the hostile/coercive parenting style only. The results of the mediation model are presented in Figure 1. Openness to different ways of thinking was negatively associated with the hostile/coercive parenting style (β = −0.278, *p* < 0.001), which was positively associated with child anxiety (β = 0.256, *p* < 0.001). The analysis confirmed the mediating role of the hostile/coercive parenting style in the association between parental openness to different ways of thinking and child anxiety (β = −0.071, CI [−0.116, −0.033]). It appears that the more parents report openness to other ways of thinking, the less they report hostile/coercive parenting style, and in turn, the less they report child anxiety.

## 5. Discussion

The purpose of the present study was to deepen our understanding of the mechanisms that contribute to the formation of child anxiety and to better understand the impact of parenting and parental characteristics on child anxiety [4,15,45]. Therefore, we examined the role of parenting styles in the association between parental openness to different ways of thinking and child anxiety. Although parenting styles have long been known to be associated with children’s outcomes [18,19,46], their underlying components have not been studied yet, specifically, cognitive processes associated with parenting styles.

The results of the present study partially confirmed the study’s hypotheses. Although the results reveal a positive association between hostile/coercive parenting and child anxiety, the hypothesized negative association between supportive/engaged parenting and child anxiety was not confirmed. The correlation between hostile/coercive parenting and child anxiety is in line with previous findings that indicate high levels of hostile and coercive behaviors among families with a variety of psychological problems [21]. Among these problems are anxiety and depression [29,30]. A hostile/coercive style is characterized with criticism, high levels of parental control, rejection, and a pessimistic world view [20], which reflect negative feelings towards the child. These emotions are often expressed by shouting, giving frequent negative commands, and expressing anger, threats, and aggression [47]. Clearly, this parent–child interaction sets the stage for the development of emotional and psychological difficulties.

Contrary to the hostile/coercive style, supportive/engaged parenting is characterized with positive feelings towards the child, acceptance of the child through affection, joint activities, and emotional as well as instrumental support [21]. According to the present findings, such a parent–child relationship does not relate to child anxiety and therefore does not prevent or reduce it. Since previous studies did not provide evidence for the association between high levels of supportive/engaged behaviors and reduced levels of child anxiety [18], the present finding strengthens our understanding of the irrelevance of parental positive components to the threat perception that underlies the development of child anxiety [18]. As indicated in previous studies, the central component that was associated with child anxiety is overcontrolled parenting [15,18].

In line with the study’s hypothesis, both parenting styles were significantly associated with parents’ openness to different ways of thinking—supportive/engaged parenting was positively associated with it, while hostile/engaged was negatively associated with it. Such a negative association between hostile/coercive parenting and parental openness to different ways of thinking has been previously established. Parents who are considered less open-minded and cognitively conservative in their views discard other points of view and consider them as flawed or illegitimate [25]. Parents who hold such a perspective tend to emphasize absolute obedience with punitive measures that characterize a hostile/coercive parenting style [29,30]. As a result, the parent may be perceived by the child as an intrusive entity that does not engage in their existence and development in an appropriate manner [15,48], which may cause great anxiety. A child’s psychological development is hindered when the parent cannot provide the environment necessary for forming a healthy sense of self, because the core of the self-authentic personality is suspended and inhibited by an adaptive obedience to the defective environment [49]. When parenting does not allow for the child to develop an independent personality with separateness and strong emotional reactions, great anxiety develops instead [50]. Therefore, the more open-minded the parent is to different ways of thinking, the less controlling and coercive the parent is towards the child and more attuned to the child’s separation and individuation, which enables the child to develop learning and emotional regulation mechanisms [51] that reduce levels of anxiety [47].

The positive association between supportive/engaged parenting style and parental openness and different ways of thinking is also consistent with the findings of previous studies. Children whose parents express open-mindedness and encourage independent thinking and self-discovery tend to develop better psychological, social, and cognitive flexibility; therefore, they have better coping mechanisms [29]. These children experience the world as a place with many opportunities to experiment within secured boundaries. Such developmental conditions of emotional wellbeing and self-concept are associated with a low probability of experiencing depression, fear, and anxiety [27,52]. Furthermore, children with good coping mechanisms and emotional control better manage situations of fear and anxiety [51].

The results of the present study confirm the mediating role of the parental hostile/coercive style in the association between openness to different ways of thinking and child anxiety. It seems that parental openness to different ways of thinking reduces the chance of adopting a hostile/coercive parenting style, which, in turn, reduces the levels of anxiety experienced by the child. Therefore, the anxiety levels of children are not directly affected by the parent’s cognitive ability for flexible thinking, but this characteristic allows for the parent to consolidate parenting that does not resort to coercive and hostile behaviors, control, obedience, and severe strictness.

## 6. Limitations of the Study

Several methodological aspects must be considered when interpreting the present findings. First, the study relied on self-report questionnaires, which might have been biased by social desirability [53]. In addition, the assessment of child anxiety was based on the parent’s reports, one of the child’s parents, which might be a limited or biased perspective. It is possible that observational studies will better reflect the anxiety levels of children. It should be noted that participants were asked to rate their answers on a 3-point Likert scale, which might have affected the potential variability of this variable and therefore impact the study’s findings. Second, we used a cross-sectional design; therefore, no causation can be inferred with certainty. It is recommended that future studies are conducted with longitudinal designs. Third, the age range of the children was 6 to 18. It is considered a wide range for examining anxiety. Future studies should consider more homogenous samples in terms of age range. Fourth, the study’s sample is gender-biased towards women. Since a stronger association exists between mothers’ parenting dimensions and child anxiety compared to fathers’ parenting [54], it is recommended that future studies use samples with an equal representation of mothers and fathers. Finally, the internal consistency of the hostile/coercive subscale was slightly lower (0.68) than recommended in the social sciences (0.7) (see, for example, [55]). Nevertheless, this difference is negligible, and the measure is widely used in the field of parenting [40].

## 7. Conclusions and Implications

Despite the study’s limitations, the current findings have important implications for practitioners, such as family therapists and parenting instructors, as well as prevention program developers who strive to improve children’s and families’ wellbeing. Professionals should be aware of the importance of parents’ cognitive skills, particularly openness to different ways of thinking, in reducing the risk of child anxiety. Professionals may assist parents to change the parental patterns of perceptions and behaviors that might increase a child’s vulnerability to developing anxiety. This can be carried out by encouraging and guiding parents to develop empathy towards their child as well as a supportive and engaged parenting style that will promote the child’s separation process and therefore promote the child’s wellbeing.

## Data Availability

Data available on request due to privacy restrictions.
